# Erastin inhibits porcine epidemic diarrhea virus replication in Vero cells

**DOI:** 10.3389/fcimb.2023.1142173

**Published:** 2023-03-01

**Authors:** Hongliang Zhang, Yingguang Li, Ruimei Yang, Ling Xiao, Shaoming Dong, Jiaxu Lin, Gang Liu, Hu Shan

**Affiliations:** ^1^ College of Veterinary Medicine, Shandong Collaborative Innovation Center for Development of Veterinary Pharmaceuticals, Qingdao Agricultural University, Qingdao, China; ^2^ Animal Husbandry and Veterinary Station of Rushanzhai Town, Rushan Animal Husbandry Development Center, Weihai, China

**Keywords:** erastin, Ferrostatin-1, porcine epidemic diarrhea virus, Ferroptosis, Vero cells

## Abstract

**Background:**

Porcine epidemic diarrhea virus (PEDV), an intestinal pathogenic coronavirus, has caused significant economic losses to the swine industry worldwide. At present, there are several treatment methods, but there is still a lack of clinically effective targeted drugs, new antiviral mechanisms and drugs need to be explored.

**Methods:**

In this study, we established a model of erastin versus ferrostatin-1 treatment of Vero cells, and then detected virus proliferation and gene expression by RT-qPCR through PEDV infection experiments.

**Results:**

We demonstrated for the first time that erastin significantly inhibited the replication of PEDV upon entry into cells; Vero treated with erastin significantly regulated the expression of three genes, *NRF2*, *ACSL4* and *GPX4*, notably erastin regulated the expression of these three genes negatively correlated with the expression induced by PEDV virus infection.

**Conclusions:**

Since *NRF2*, *ACSL4* and *GPX4* are classical Ferroptosis genes, this study speculates that erastin may inhibit the replication of PEDV in Vero cells in part through the regulation of ferroptosis pathway, and erastin may be a potential drug for the treatment of PEDV infection.

## 1 Introduction

Porcine epidemic diarrhea virus (PEDV), a member of the *Coronaviridae* family, has an enveloped single-stranded RNA genome of approximately 28.5 kb in size that primarily infects porcine small intestinal epithelial cells, and is capable of replicating in Vero cells to trigger changes in host gene expression patterns (Xu et al., 2019; Wang et al., 2022a). Among the four structural proteins of the PEDV genome, the spike (S) glycoprotein consists of S1 and S2 subunits, which acts as a PEDV surface antigen to regulate the interaction between host cell receptor proteins and the virus, mediating the entry of the virus into the cell (Makadiya et al., 2016; Wang et al., 2022a). During PEDV infection of cells, the expression of surface transferrin receptor 1 (TFR1) in enterocytes, the changes in the expression level of transferrin receptor 1 (TFR1) on the surface of enterocytes, andthe disruption of cellular iron homeostasis by citrulline and desferrioxamine have implications for PEDV proliferation in cells (Zhang et al., 2020). Therefore, as a key factor that may regulate the proliferation of PEDV, iron has the value of in-depth research.

Iron plays an important role in the replication of many viruses and is also important in maintaining cellular physiology (Dartois and Rubin, 2022). The enterocyte pathway is a key factor in iron uptake by organisms and maintenance of iron homeostasis (Silva and Faustino, 2015). A variety of viruses have been found to interrupt iron uptake and antioxidant response systems, while some viruses use iron transporter proteins as receptors to mediate cell invasion (Wang et al., 2022b). Ammonium ferric citrate as an iron supplement to promote membrane fusion and inhibit endosome release in IAV, HIV, ZIKV, and Enterovirus 71 (EV71) (Wang et al., 2018b). Levels of serum proteins tend to remain high for long periods in HIV-infected patients (Drakesmith and Prentice, 2008). Infection with swine flu virus disrupts the dynamic balance of intracellular iron and redox. Inhibition of the system Xc-/*GPX4* axis leads to cellular ferroptosis (Cheng et al., 2022). The occurrence of ferroptosis during the proliferation cycle of PEDV thus becomes a focus of our attention.

Ferroptosis is a non-apoptotic form of cell death that relies on accumulation of intracellular iron and causes elevated toxic lipid peroxides Reactive oxygen species (ROS) (Dixon et al., 2012; Latunde-Dada, 2017). Erastin, a classical activator of ferroptosis, triggers ferroptosis by inhibiting xc-system-mediated cystine uptake. (*1S,3R*)*-RSL3* is an inhibitor of glutathione Peroxidase 4 (GPX4) (ferroptosis agonist)*. RSL3* targets downstream of the xc-system and directly inhibits *GPX4* to trigger the ferroptosis process (Yagoda et al., 2007; Dixon et al., 2012; Wang et al., 2018a). Ferrostatin-1 (Fer-1) is a synthetic antioxidant that effectively inhibits RSL3 induced death and inhibits the function of erastin (Yagoda et al., 2007; Dixon et al., 2012; Skouta et al., 2014; Zhou et al., 2020). Two compounds were used as classical ferroptosis pathway activators and inhibitors to intervene in the PEDV infection process of Vero cells, in order to observe the effect of PEDV regulating cellular iron death pathway during Vero infection on PEDV infection.

In this study, we aimed to reveal the effect of erastin on the process of PEDV infection of Vero cells. Modeling of cellular ferroptosis pathway regulation was established by erastin and Fer-1 treatment of Vero cells; PEDV infection of Vero cells was performed under this variable. This study is innovative to carry out relevant studies in the ferroptosis pathway of porcine epidemic diarrhea virus, laying a scientific foundation for the development of potential iron metabolism drugs antiviral drugs.

## 2 Materials and methods

### 2.1 Cells, viruses and reagents

Vero cells (maintained in the Shandong Key Lab of Preventive Veterinary Medicine, Qingdao Agricultural University) were cultured in DMEM high glycemic liquid medium (HyClone Laboratories, Inc., Logan, UT) with 10% fetal bovine serum (VivaCell, Shanghai, China). The maintenance medium consisted of DMEM high glycemic liquid medium supplemented with 12.5 μg/mL trypsin (Gibco, USA) without the addition of serum. Briefly, the fused cell monolayers were washed once with sterile phosphate-buffered saline (PBS) and infected with maintenance medium supplemented with 12.5 μg/mL trypsin with PEDV SD2020 (GenBank OP894120) for 1 hour at 37°C; then, the viral solution was removed, the cells were washed twice with PBS, and maintenance medium was added. Harvest the cell cultures until the cytopathic effect (CPE) exceeds 80%. After freeze-thaw processing, supernatants were collected and stored at -80°C until required (Shi et al., 2017). Rabbit anti-PEDV spike antibody was generated by the laboratory (Shandong Key Lab of Preventive Veterinary Medicine, Qingdao Agricultural University), and FITC goat anti-rabbit antibody was purchased from Wanleibio Co., Ltd (Shenyang, China).

### 2.2 Cell viability assay

Cell Counting Kit-8 (CCK-8) (Beyotime Biotech, China) was used to measure the corresponding optical density ratio marker cell viability levels in 96-well plates according to the manufacturer’s instructions. Briefly, Vero cells were resuspended in fresh medium at a density of 1×10^4^ cells/mL, 100 µl of cell suspension was transferred into 96-well microplates, and preincubated in a humidified incubator (37°C, 5% CO_2_) for 12 h. Erastin (2-29 µM), DMSO (2-29 µL/mL), Fer-1 (2-180 µM) were then added. After 18 hours incubation, 10 μL of CCK-8 solution was added to each well of the plate and further incubated for 2 hours protected from light. Levels of labeled cell viability were calculated by measuring absorbance at 450 nm using a microplate reader (Tecan, Mannedorf, Switzerland) (Yuan et al., 2021).

### 2.3 Detection of reactive oxygen species

ROS levels were measured using a reactive oxygen species assay kit (Beyotime Biotech, China). Briefly, Vero cells adhered at a density of 1×10^4^ cells/mL in 96 microtiter plates, and ROS levels were measured after 6 hours of treatment with erastin (8 μM) supplied with Fer-1 (60 μM), fer-1 (60 μM), erastin (8 μM), and a blank control. ROS level experimental groups treated for 12 hours were also done. Samples were collected according to the kit method, and cells in 96-well plates were washed twice with potassium-buffered saline (PBS) and incubated at a final concentration of 10 μM DCFH-DA for 30 min at 37°C. Excess DCFH-DA was removed by washing three times with serum-free culture medium. Using a microplate reader at 488 nm excitation wavelength and 525 nm emission wavelength, the intensity of sample fluorescence was detected in real time, and changes in intracellular reactive oxygen species content were determined by comparing with the blank group (Yuan et al., 2021).

### 2.4 Lipid peroxidation assay

Lipid peroxidation was determined by measuring the levels of malondialdehyde (MDA) as described in the Lipid Peroxidation MDA Detection Kit (Beyotime Biotech, China). Briefly, samples were grouped as in 2.3, and after 6 or 12 hours of treatment, cells were washed three times with PBS, and the supernatant was homogenized by lysing cells with NP-40 (Beyotime Biotech, China) according to the manufacturer’s instructions. Then, the TBA-MDA mixture was heated to 100°C for 60 min. The absorbance of the mixture was measured by an enzyme marker at 532 nm, and calculated in μM/mg protein based on the concentration of the standard MDA (Kan et al., 2021).

### 2.5 Quantitative Real-Time PCR

Total RNA was extracted from cells using the SteadyPure Universal RNA Extraction Kit (Accurate Biotechnology Co., Ltd., Hunan, China) according to the manufacturer’s instructions. First-strand cDNA synthesis was performed using HiScript^®^ II Q RT SuperMix for qPCR (Vazyme Biotech co., Ltd, China). Taq Pro Universal SYBR qPCR Master Mix (Vazyme Biotech co., Ltd, China) reagents were used for dye fluorescent PCR assays using the Bio-Rad CFX Connect Real-Time System (Applied Biosystems™ Quant Studio 5 Real-Time PCR System, Thermo Fisher Scientific). The RT-qPCR reaction volume was 20 μL, which included 10 μL 2 × SYBR^®^ Premix Ex Taq (Takara Bio, Inc., Kusatsu, Japan), 1 μL (10 pmol/L) forward primer, 1 μL (10 pmol/L) reverse primer, 2 μL template DNA and 6 μL sterile water, primer sequence as shown in **Table 1**. Reaction conditions were as follows: denaturation for 30 s, followed by 40 cycles of 95°C for 30 s, 60°C for 30 s, 72°C for 30 s, and finally melting curve determination. Calculations were performed with reference to the relative quantitative analysis (2^-ΔΔCt^) method (Shi et al., 2017; Lu et al., 2018; Xu et al., 2020).

**Table 1:** Primers for real-time PCR.

Primer name	Nucleotide sequence (5’-3’)	Product (bp)	GenBank accession number
PEDV N	F: ACTAATAAAGGGAATAAGGACCAG	207	OP894120
	R: GTTAGTGGGTTCAGTCTTTGC		
ACSL4	F: AATAGACATCCCTGGAGCAGATACT	119	NM_001318509.2
	R: GCTGCATTTCATTTTCTTCACTTAG		
NRF2	F: TCCAGTCAGAAACCAGTGGAT	109	HM446346.1
	R: GAATGTCTGCGCCAAAAGCTG		
GPX4	F: CAGTGAGGCAAGACCGAAGTGAAC	125	NM_001039847.3
	R: TTACTCCCTGGCTCCTGCTTCC		
GAPDH	F: CCCACTCCTCCACCTTTGAC	113	NM_002046.7
	R: TCCACCACCCTGTTGCTGTAGC		

### 2.6 Indirect immunofluorescence

For cell culture, monolayers of cells were grown in 48-well plates, grouped and treated after the cells were rinsed three times with PBS, and fixed with 4% formaldehyde for 15 min, followed by three washes with PBS. Cells were penetrated into the solid solution using 0.1% Triton X-100 on ice for 5 min, followed by 3 washes with PBS. The cells were closed using Immunofluorescence Fast Blocking Solution for 30 min, and the primary antibody was incubated for 3 hours with the addition of rabbit anti-PEDV prickle protein polyclonal antibody. The cells were then incubated with FITC goat anti-rabbit antibody for 37°C for 1 hour. Cells were then washed 3 times with PBS, then washed with DIPA staining solution for 5 min, followed by glycerol blocking, and then observed using a fluorescence microscope (Xu et al., 2020).

### 2.7 Statistical analysis

Statistical analysis and graph production were performed using GraphPad Prism (GraphPad Software) version 6, and Microsoft Excel was used to perform ANOVA for data analysis. All data are expressed as mean ± standard deviation. *P* < 0.05 were considered statistically significant, while *P* < 0.01 was considered highly significant.

## 3 Results

### 3.1 Evaluation of the cytotoxicity of erastin and Fer-1

**Figure 1:**
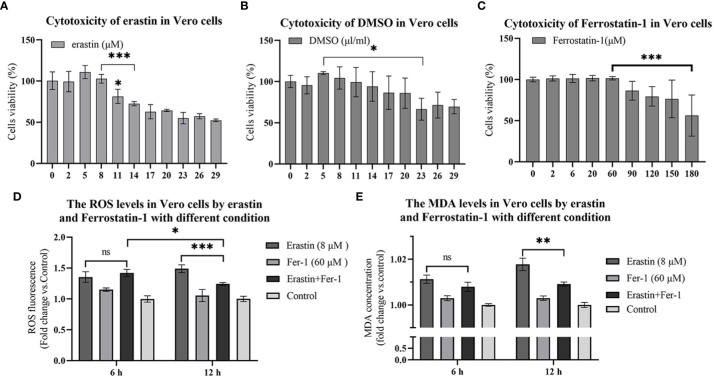
16 hours cytotoxicity of Vero cells. ROS and lipid oxidation levels were used to evaluate the biological functions of erastin and Fer-1 in Vero cells. **(A–C)** Cytotoxicity of erastin, Ferrostatin-1 and DMSO in Vero cells at 16 hours. **(D)** The ROS levels of Vero cells treated with erastin and Fer-1 were detected at 6 h and 12 h. **(E)** Malondialdehyde levels in Vero cells treated with erastin and Fer-1 were detected at 6 and 12 hours. The data were performed from three independent experiments, with the untreated group as the control group. The differences were evaluated using ANOVA test. The error bar represents the standard deviation. ns, not significant, * *P*<0.05, ** *P*<0.01, *** *P*<0.001.

To investigate the potential cytotoxicity of erastin, Fer-1 and solvent DMSO, the relative survival of erastin, Fer-1 and DMSO treated Vero cells after 16 hours was tested by CCK-8 assay. The erastin concentrations ranged from 2 μM to 20 μM, DMSO concentrations ranged from 2 μl to 20 μl, and Fer-1 concentrations ranged from 2μM to 180 μM, respectively. The cytotoxic effect of erastin was dose-dependent, with erastin (11 μM) showing a decrease in cell viability, and 14 μM showed a significant decrease in cell viability compared with 8 μM and below (**Figure 1A**). DMSO showed a decrease in cell viability at concentrations of 20 μl/ml and significant cell damage at concentrations above 23 μl/ml (**Figure 1B**). Fer-1 had no significant damaging effect on cells at cytotoxic concentrations below 60 μM (**Figure 1C**). Therefore, subsequent assays were performed using concentrations of erastin below 8 μM and Fer-1 below 60 μM.

### 3.2 Validation of erastin and Fer-1 activation and inhibition of lipid oxidation in Vero cells

Ferroptosis is mainly characterized by intracellular iron overload and redox imbalance. When ferroptosis occurs, intracellular iron overload can lead to ROS overproduction through the Fenton reaction. Therefore, ROS accumulation and mitochondria lipid oxidation are closely related to ferroptosis. Therefore, oxidative stress and lipid peroxidation assays were performed to confirm the generation of ferroptosis. Vero cells were assayed for levels of cellular reactive oxygen species after erastin and Fer-1 treatment using the ROS reactive oxygen species assay kit. In 6 hours treatment group, erastin (8 μM) was not significantly different from erastin (8 μM) and Fer-1 (60 μM) co-treatment group, while erastin (8 μM) ROS levels were significantly higher than in the erastin (8 μM) and Fer-1 (60 μM) co-treatment group at 12 hours (*P <*0.001) (**Figure 1D**). Analyzing the reactive oxygen levels in Vero cells at 6 hours versus 12 hours treatment, erastin (8 μM) and Fer-1 (60 μM) co-treatment groups significantly decreased at 12 hours (*P <*0.05) (**Figure 1D**). The above results could indicate that erastin and Fer-1 were able to activate intracellular reactive oxygen species production in Vero cells treated with erastin, while Fer-1 could inhibit this process at 12 hours but was less potent than erastin.

Malondialdehyde (MDA) is a product of lipid oxidative catabolism when cells undergo oxidative stress. Vero cell lipid peroxidation levels after erastin and Fer-1 treatment were detected by MDA to verify the production of ferroptosis. Erastin (8 μM) and Fer-1 (60 μM) co-treatment groups in the comparison between the two groups at 6 hours and 12 hours were significantly decreased (*P*<0.01) (**Figure 1E**), and this result was similar to the ROS level assay. Based on this model, we added erastin and Fer-1 to PEDV-infected Vero cells to observe their regulatory effects on PEDV proliferation.

### 3.3 Proliferation of PEDV virus in Vero cells is influenced by erastin and Fer-1

**Figure 2:**
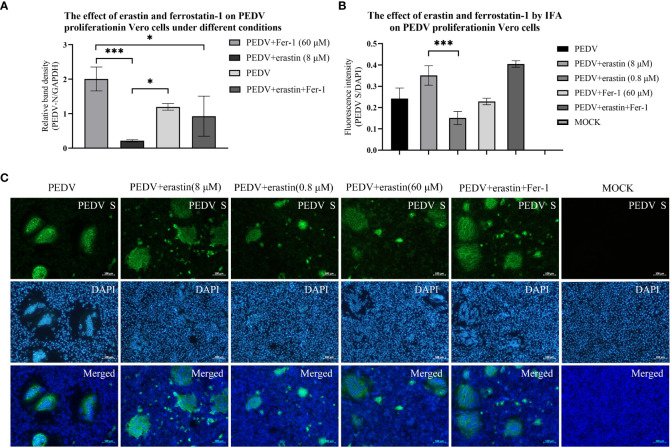
Effects of erastin and Fer-1 on Vero cells infected by PEDV. **(A)** Relative quantification of PEDV for verification by RT-qPCR. The relative quantification of PEDV in Vero cells with different treatments by RT-qPCR was calculated by 2^-ΔΔCt^ method. **(B)** The Analyze the fluorescence intensity of virus-infected cells in each of experiments by using the ImageJ software. Error bars represent standard deviations of technical triplicates. *P<0.05, ***P < 0.001, n = 3 per group. **(C)** Vero cell with different treatments were collected at 10 hours, fixed, permeabilized, and stained with PEDV S protein (rabbit), then rabbit protein antibody (goat). DAPI was used for nuclear staining. Images were taken using a microscope. Scale bar = 100 μm.

The effect of erastin and Fer-1 on the proliferation of PEDV in Vero cells was investigated by RT-qPCR with rabbit polyclonal antibody IFA to PEDV spiked protein. During the infection of 10 MOL PEDV virus in Vero cells, the addition into the maintenance solution was performed using erastin (8 μM) and Fer-1 (60 μM), respectively, and the total intracellular RNA of the samples was extracted for PEDV nucleic acid copy number at the appearance of lesions in Vero cells after 12 hours. The addition of erastin (8 μM) treated group showed decreased differences compared to the PEDV infected group (*P*<0.05), while the Fer-1 (60 μM) treated group showed highly significant differences compared to the erastin (8 μM) and Fer-1 (60 μM) co-treated groups (*P*<0.05), especially the PEDV nucleotide content was highly significantly reduced in the erastin (8 μM) treated group compared to the Fer-1 (60 μM) treated group (*P*<0.001) (**Figure 2A**). Based on this result, we concluded that erastin can inhibit the proliferative effect of PEDV in Vero cells, while Fer-1 can partially inhibit erastin from exerting this biological function.

IFA presented the results of Vero cells infected with PEDV lesions, and cells were treated by adding erastin (8 μM and 0.8 μM) and Fer-1 (60 μM) into maintenance medium. Samples were fixed at 10 hours when significant lesions developed in the PEDV infected group. Cells supplemented with erastin (8 μM) produced significantly higher CPE results compared with wells treated with erastin (0.8 μM), and high concentrations of erastin increased cell damage, while Fer-1 (60 μM) did not show substantial CPE consistent with RT-qPCR results (**Figure 2B**). And we observed that the lesions presented by the test group in which erastin (8 μM) was co-added to the maintenance medium with Fer-1 (60 μM) were the most significant (**Figure 2C**). Based on the RT-qPCR data and IFA observations described above, we suggest that the biological function of erastin in Vero cells would inhibit the proliferation of PEDV in Vero cells, but its high concentration was also able to induce lesions in cells. Fer-1, on the other hand, did not significantly inhibit cellular lesions, and based on this we further investigated the process by which erastin inhibits PEDV.

### 3.4 Erastin affects the adhesion and invasion of PEDV virus on Vero cells

**Figure 3:**
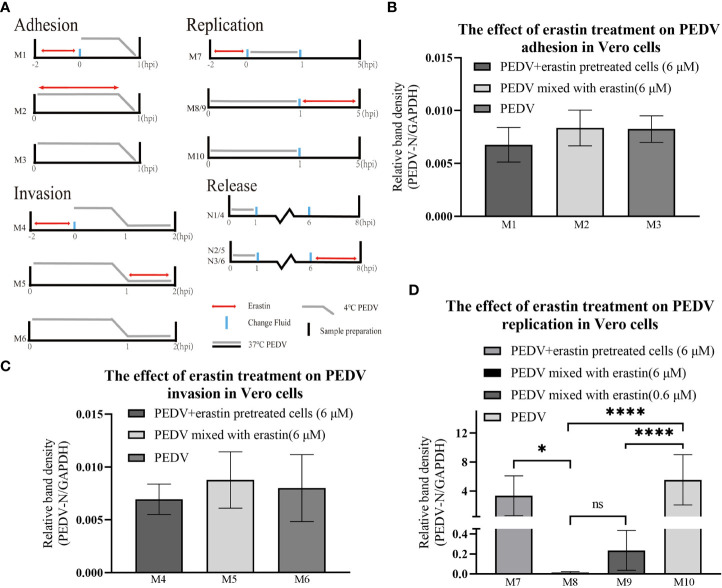
Erastin affects the different stages of PEDV infection with Vero cell. **(A)** Vero cells or PEDV were treated with erastin in different ways before and after infection. Double-headed red arrows indicate the presence of erastin. The gray line indicates the PEDV infection. The experiments are identified in the text by the numbers on the left (M1-M10) and (N1-N6). Cell treatment time is calculated as hour post infection below. The relative quantity of RT-qPCR of PEDV in Vero cells treated with erastin was calculated by 2^-ΔΔCt^ method. **(B–D)** The relative quantity of PEDV in Vero cells at the stages of adhesion, invasion and replication was verified by RT-qPCR. The error bar represents the standard deviation * *P*<0.05, **** *P*<0.001, 3 cases in each group. ns, not significant.

In this study, the effect of erastin on the proliferation stage of PEDV infected Vero was further investigated, and the effect of erastin on the viral replication cycle was determined by adding erastin groups in four stages: PEDV adhesion, invasion, replication, and release (**Figure 3A**). Adherence assays were performed by infecting Vero cells with PEDV and washing the total cellular RNA after incubation at 4°C for 1 hour to obtain viral nucleic acid copy number. The grouping of adhesion and invasion experiments is (M1-M6) shown in **Figure 3A**. results are shown in **Figures 3B, C**. Treatment of Vero cells with erastin had no significant effect on PEDV adhesion and invasion into Vero. Based on this result we focused our study on the effect of erastin on replication.

### 3.5 Erastin affects the replication of PEDV virus in Vero cells

To determine the effect of erastin on viral replication, the experiment was divided into different groups (**Figure 3A**). After PEDV infection of Vero cells at 37°C for 1 hour, the cells were washed and the maintenance medium was changed for each group, and the total intracellular RNA was extracted at 5 hours to analyze the effect of viral replication. M8 and M9 showed highly significant downregulation (*P*<0.001) compared with M10, even though the concentration of erastin (0.6 μM) in M9 was 10 times more diluted than that of erastin (6 μM) in M8; while group M7 pretreatment showed significant difference (*P*<0.05) compared with group M8, but no significant difference with M10 (**Figure 3D**). The above results demonstrate that erastin exerts an inhibitory effect during intracellular replication of PEDV, even after 10-fold dilution. Erastin pretreatment experiments demonstrate that its inhibitory effect decreases after discontinuation of drug administration.

**Figure 4:**
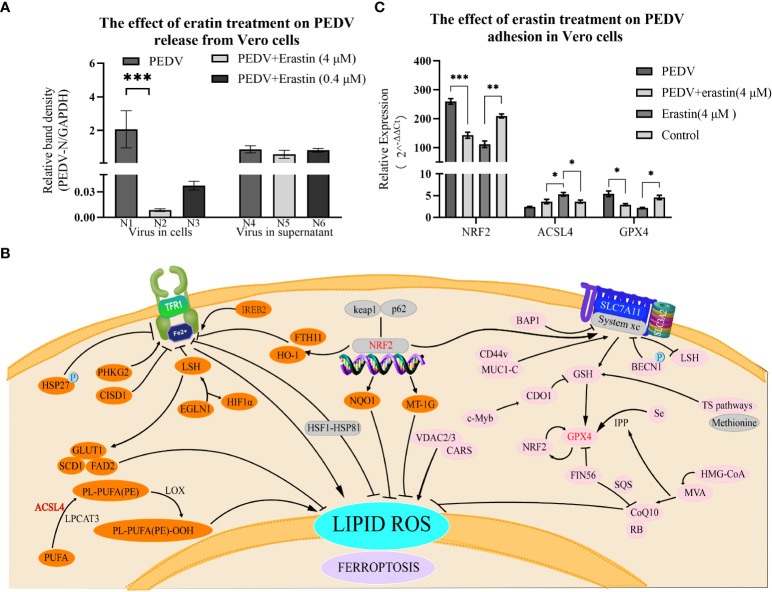
Erastin affects the release of Vero cells infected with PEDV and the regulation of ferrotropis gene. **(A)** The relative quantity of PEDV in Vero cells at the release phase was verified by RT-qPCR. The left part (N1-N3) represents the virus in cells, and the right part (N4-N6) represents the virus in the supernatant. **(B)** Ferroptosis-suppressing pathways. *NRF2*, *ACSL4* and *GPX4* are key factors in the iron death signaling pathways of P53-*NRF2* - (*HO-1/NQO1*), PUFA *ACSL4* PUFA-PL and system Xc-*GSH*-*GPX4*. **(C)** The expression of *NRF2*, *ACSL4* and *GPX4* was determined by RT-qPCR, GAPDH was the loading control. Cell treatment time is calculated as per hours after infection, 2^-ΔΔCt^ method was used to calculate the relative quantity of RT-qPCR of PEDV in Vero cells treated with Erastin. The error bar represents the standard deviation *P<0.05, **P<0.01, ***P<0.001,3 cases in each group.

To determine the effect of erastin on virus release. Vero cells were infected with PEDV and the maintenance medium was replaced for 5 hours followed by erastin-supplemented medium, and the supernatant and pellet were collected at 37°C for 2 hours to extract viral supernatant nucleic acid to assess the release of PEDV and extract intracellular RNA to assess the content in viral cells. The results of cell precipitation were similar to replication assessment N2 and N3 showed highly significant differences from N1, respectively (*P*<0.001) (**Figure 4A**). While the differences between the viral content released in the supernatant N4, N5 and N6 were not significant. In conclusion, these results indicate that erastin is able to perform the biological function of inhibiting viral replication in the process of Vero infection with PEDV, has no significant effect on the release of the virus, and can effectively inhibit the replication process of the virus in the cells.

### 3.6 Regulation of *NRF2*, *ACSL4* and *GPX4* by erastin and PEDV in Vero Cells

To verify whether erastin regulates ferroptosis-related genes during PEDV infection replication, *NRF2*, *ACSL4* and *GPX*4 genes were selected to detect the regulatory levels (**Figure 4B**). Erastin-treated Vero cells significantly up-regulated *ACSL4* and significantly down-regulated *NRF2* and *GPX4* gene expression levels; comparing the PEDV-infected group with the erastin-treated PEDV-infected group based on the blank cell group, PEDV infection up-regulated *NRF2* and *GPX4*, and down-regulated *ACSL4* (**Figure 4C**). The *NRF2* gene was significantly down-regulated by erastin (4 μM) compared to Control (*P*<0.001); the PEDV infection group significantly up-regulated *NRF2* gene expression compared to Control (*P*<0.001), while the cellular difference was highly significant in the PEDV infection group compared to erastin treatment (*P*<0.0001). there was a significant down-regulation of *ACSL4* expression in the PEDV infected group compared to erastin (4 μM) (*P*<0.0001), while there was a down-regulation compared to Control (*P*<0.05). Similarly, erastin had significant up-regulation of *ACSL4* gene expression compared to Control (p<0.05). *GPX4* gene was down-regulated in erastin group compared to Control, while PEDV infected group was significantly upregulated compared to Control (*P*<0.05). Similarly, erastin-treated PEDV infection was significantly down-regulated compared to PEDV-infected group (*P*<0.05). Taken together, the regulation of *NRF2*, *ACSL4* and *GPX4* genes by erastin in Vero cells proved to be stimulating the onset of ferroptosis, while the regulation in the PEDV-infected group was opposite to the onset of ferroptosis. The regulation of PEDV-infected gene expression was pulled back by erastin treatment while the manifestation showed that erastin inhibited PEDV replication in Vero cells. Based on this phenomenon, we speculate that the regulation of the cellular ferroptosis pathway by erastin would have an effect on the replication process of PEDV. Since intracellular replication of porcine epidemic diarrhea virus is a complex process, the relevant results still need to be corroborated by more subsequent studies.

## 4 Discussion

Vaccination is currently an effective method applied to the clinical control and eradication of PEDV infection, mainly by giving piglets protection against PEDV through maternal antibodies (Langel et al., 2020). Although several compounds currently exhibit antiviral activity in PEDV-infected Vero cells, this is the first time that PEDV-infected Vero cells have been studied with ferroptosis-related drugs. In this study, we demonstrated that the classical inducer of ferroptosis regulation, erastin, and the inhibitor, Fer-1, have effects on the proliferation of PEDV-infected Vero cells. The regulation of ferroptosis-related genes after PEDV virus infection of cells is consistent with the study of the trend in the outcome of PEDV-infected piglets in the inhibition of transferrin receptor 1 (Zhang et al., 2020). Compared with Vero, intestinal cells can more realistically present the relationship between drugs, viruses, and organisms. However, based on the experience of PEDV infection of IPEC-J2 intestinal cells in our laboratory and the differences in the sensitivity of IPEC-J2 cells to PEDV proved in the literature, Vero cells can better reduce the differences of PEDV infection among samples. Vero, as a susceptible cell of PEDV virus, has also been widely used in the mechanism research of PEDV. For the above reasons, Vero cells were selected as the *in vitro* model in this study.

As a common pathway in viral infection of cells, various viruses have been found to interrupt the iron metabolism system or use related receptors to complete the infection (Drakesmith and Prentice, 2008). Iron also has a clear antiviral function in influenza virus (IAV), human immunodeficiency virus (HIV), Zika virus (ZIKV), etc (Wang et al., 2022b). Based on the existing studies on the expression level of cell surface transferrin receptor-1 and infection by PEDV virus (Zhang et al., 2020). In this study, the classical activator of ferroptosis, erastin, and the selective inhibitor of ferroptosis, Fer-1, were chosen to achieve the regulation of cellular self-reactive oxygen species and iron levels (Wang et al., 2022b). In this study, erastin and Fer-1 were chosen to achieve regulation of cellular self-reactive oxygen species and iron levels. In this study, erastin was found to induce reactive oxygen species activation and lipid oxidation in Vero cells to perform biological functions, while being able to interfere with the proliferation process of the virus on the cell. According to the coronavirus life cycle, the four phases of adhesion, invasion, replication, and release were validated (Fehr and Perlman, 2015; Xu et al., 2020). Erastin was found to be able to inhibit extremely significantly the replication of the virus in the cell, although the process ultimately failed to interrupt the cell death process. In the present study, it was shown that Fer-1 can inhibit part of the biological functions of erastin in PEDV-infected Vero cells. This process is similar to previous studies in which ferric citrate inhibited PEDV infection in primary small intestinal cells (Zhang et al., 2020).

In the selection of ferroptosis-related genes, *NRF2*, *ACSL4* and *GPX4* were selected as observation genes in this study. Erastin is capable of triggering ferroptosis in several ways, such as the ability to upregulate SLC7A11 and indirectly downregulate intracellular glutathione levels (Dixon et al., 2012), reversal of microtubulin inhibition of VDAC (DeHart et al., 2018). *NRF2* can directly or indirectly regulate *GPX4* expression and function by increasing the expression of target genes related to iron and ROS metabolism, such as quinone oxidoreductase 1 (NQO1), HO-1 and System Xc- (Jiang et al., 2015; Abdalkader et al., 2018; Song and Long, 2020). *ACSL4* is a key enzyme for the synthesis of PUFAs, the raw material for lipid peroxidation, a key component of ferroptosis, and a key target for antiviral activity (e.g., **Figure 4B**) (Kung et al., 2022). *GPX4* is a peroxide reductase and a key node in the biological function of erastin, and its downregulation can lead to the accumulation of lipid peroxides (Maiorino et al., 2018). In this study of erastin, the expression of the genes *NRF2*, *GPX4* and *ACSL4* were evaluated for regulation, and erastin treatment of Vero cells exerted its ferroptosis-inducing regulatory role (Abdalkader et al., 2018; Lu et al., 2018; Kan et al., 2021). It is interesting to note that the regulation of gene expression in Vero cells by PEDV infection is opposite to the occurrence of ferroptosis, and that erastin intervention in PEDV replication in turn regulates gene expression.

In conclusion, erastin activates reactive oxygen species and lipid oxidation in Vero cells, while Fer-1 inhibits this process. During PEDV infection of Vero, the addition of erastin inhibits the replication of PEDV in cells, but has no significant effect on viral adhesion, invasion and release. Erastin-induced regulation was inversely correlated. Further studies on the targets of the ferroptosis pathway of PEDV virus can be carried out, and the potential antiviral capabilities with ferroptosis-related drugs such as erastin can be further developed.

## Data availability statement

The original contributions presented in the study are included in the article/supplementary material. Further inquiries can be directed to the corresponding authors.

## Author contributions

HS and GL conceived the idea. HZ and YL did most of the experimental work and wrote the first draft of the manuscript. RY and LX collected all the data. SD and GL advised in the process of manuscript writing. All authors contributed to the article and approved the submitted version. All authors agreed to be accountable for the content of the work.
